# Targetable Effects of the Anesthetic, Ubiquinone‐5, on Murine Cardiac Rhythm

**DOI:** 10.1096/fj.202504065RR

**Published:** 2026-02-16

**Authors:** Haeun Lim, Rong Lu, Chloe Shi, Richard J. Levy

**Affiliations:** ^1^ Department of Anesthesiology Columbia University Irving Medical Center New York USA; ^2^ University of Missouri‐Kansas City School of Medicine Kansas City Missouri USA

**Keywords:** anesthesia, Aralar, arrhythmia, cardiotoxicity, mitochondria, ubiquinone

## Abstract

General anesthetics can adversely affect the heart, negatively impacting chronotropy, electrical conduction, and myocardial contractility. The intravenous sedative‐hypnotic, propofol, for example, impairs ventricular contraction at clinically relevant doses and can cause dysrhythmias and atrioventricular block with acute administration. In addition, high cumulative propofol doses can induce bradyarrhythmias, cardiac conduction abnormalities, and myocardial failure. As with propofol, the recently identified intravenous anesthetic agent, ubiquinone‐5 (Ub5), causes bradycardia and complete heart block at supratherapeutic doses. However, the cardiac effects of clinically relevant Ub5 doses are unknown. Thus, we aimed to determine how therapeutic doses of Ub5 impact cardiac rhythm, hypothesizing that Ub5 would interfere with dromotropy. We tested our hypothesis in vivo in the young adult mouse and ex vivo in the isolated‐perfused murine heart. We then determined mechanistic contributors of Ub5‐induced cardiotoxicity in isolated cardiomyocyte mitochondria. We found that Ub5 caused type 1 s‐degree heart block and compromised the mitochondrial membrane potential in isolated cardiomyocyte mitochondria by inhibiting electron transport and inducing excessive proton leak. Pharmacological inhibition of the aspartate–glutamate carrier, Aralar, rescued Ub5‐mediated disturbances in cardiac rhythm in the isolated‐perfused heart. The findings suggest that Ub5 can impact cardiac conduction in a targetable manner, carrying importance for future drug development efforts.

## Graphical abstract

The novel anesthetic, ubiquinone‐5 (Ub5), causes second‐degree heart block, in part, by compromising the mitochondrial membrane potential (ΔΨ) in cardiomyocytes. Ub5‐mediated inhibition of electron transport and induction of excessive proton leak via the aspartate–glutamate carrier, Aralar, prevent mitochondria from generating and maintaining an adequate ΔΨ. Inhibition of Aralar can rescue cardiac rhythm, suggesting that Ub5‐induced cardiotoxicity is targetable.

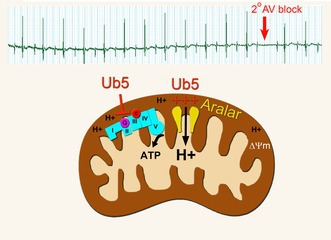

## 1 Introduction

General anesthetics are a distinct class of drugs capable of inducing reversible unconsciousness [1, 2]. Although their primary pharmacological targets reside within the central nervous system, anesthetics can have “off‐target” effects on the heart, negatively impacting chronotropy, electrical conduction, and myocardial contractility [3, 4]. Such side effects are drug‐specific and can be dose‐dependent [3, 5, 6, 7]. Propofol, the most widely used intravenous sedative‐hypnotic, commonly impairs ventricular contraction and results in vasodilation at clinically relevant doses and occasionally causes dysrhythmias and atrioventricular (AV) block following acute administration [8, 9, 10, 11]. In addition, long‐term continuous administration of high doses of propofol can induce bradyarrhythmias, cardiac conduction abnormalities, and myocardial failure in the critically ill [12, 13, 14]. Such life‐threatening cardiotoxicity is seen as a manifestation of propofol infusion syndrome, especially in infants and children [12, 13, 14].

Propofol is thought to induce overt cardiotoxicity when high concentrations of the drug interfere with the ability of cardiomyocyte mitochondria to generate and maintain the mitochondrial membrane potential (ΔΨm) [14, 15, 16, 17, 18, 19]. Recent investigation revealed that the coenzyme Q analog, ubiquinone‐5 (Ub5), exerts similar biological activity within mitochondria and acts as an anesthetic when injected intravenously [20]. As with propofol, supratherapeutic doses of Ub5 cause reversible bradycardia and complete heart block in the isolated‐perfused newborn mouse heart [16, 20]. However, the cardiac effects of clinically relevant Ub5 doses have not yet been assessed. Side effects of novel anesthetics need to be evaluated as part of the drug development process. Thus, in this work, we aimed to determine how therapeutic doses of Ub5 impact cardiac rhythm, hypothesizing that Ub5 would interfere with dromotropy. We tested our hypothesis in vivo in the young adult mouse and ex vivo in the isolated‐perfused murine heart. We then attempted to define mechanistic contributors of Ub5‐induced cardiotoxicity in isolated cardiomyocyte mitochondria.

## 2 Materials and Methods

### 2.1 Animal Ethics

Care of the animals in this study was in accordance with National Institutes of Health, Columbia University Irving Medical Center Institutional Animal Care and Use Committee (IACUC), and ARRIVE guidelines and conformed to the provisions of the Animal Welfare Act (NIH/DHHS) and the Association for Assessment and Accreditation of Laboratory Animal Care (AAALAC). The experimental protocol was approved by the Columbia University Irving Medical Center IACUC.

### 2.2 Animals

C57BL/6 male mice were acquired from Charles River (Wilmington, MA) and were studied at 6–12 weeks of age to model for a timepoint in young adulthood. Mice with a heterozygotic mutation of the *Aralar* (*Slc25a12*) gene on a SVJ129 × C57BL/6 background were acquired from Taconic Biosciences (Germantown, NY) and mated to yield *Aralar* KO (*Aralar* ^*−/−*^) mice and wild‐type (*Aralar* ^*+/+*^) littermate controls. Genotype was determined by standard PCR. *Aralar* ^*−/−*^ and *Aralar* ^*+/+*^ male mice were studied at 10 days of life.

### 2.3 In Vivo Injection

Mice were randomly injected via tail vein with a single dose of ubiquinone‐5 (50 mg/kg, 100 mg/kg, or 200 mg/kg) or equal volume of vehicle (intralipid). Electrocardiogram (ECG) was monitored using needle electrodes (ADInstruments, Castle Hill, Australia) and heart rate and rhythm were continuously recorded using an analog‐to‐digital converter system (Power Lab 4SP, ADInstruments, Castle Hill, Australia).

### 2.4 Isolated‐Perfused Mouse Heart Preparation

Mice were anesthetized (pentobarbital, 70 mg/kg i.p.) and heparinized (10 kU/kg i.p.). The heart was excised and aorta was cannulated as described [21]. Constant flow retrograde perfusion was initiated (5 mL·min^−1^) with modified Krebs–Henseleit buffer containing (mmol/L): NaCl 120, KCl 4.7, MgSO_4_ 1.2, KH_2_PO_4_ 1.2, CaCl_2_ 1.25, NaHCO_3_ 25, and glucose 11. Non‐recirculating buffer equilibrated with 95% O_2_‐5% CO_2_ was maintained at pH 7.4 and 37°C. Following stabilization, ECG was captured using surface electrodes. Heart rate and rhythm and aortic perfusion pressure were continuously recorded using an analog‐to‐digital converter system (Power Lab 4SP, ADInstruments, Castle Hill, Australia). Exclusion criteria were strictly enforced as previously described [22]. Hearts were then exposed to ubiquinone‐5 (200 mg/kg heart weight) or an equal volume of intralipid. For rescue experiments, hearts were exposed continuously to ubiquinone‐5 (200 mg • kg heart weight^−1^ • minute^−1^) or equal volume intralipid with subsequent continuous co‐administration of the Aralar inhibitor, pyridoxal 5′‐phosphate (PLP)(400 mg • kg heart weight^−1^ • minute^−1^). In separate experiments, PLP (400 mg • kg heart weight^−1^ • minute^−1^) was continuously administered alone.

### 2.5 Isolation of Mitochondria

Cardiac ventricles were harvested and homogenized in ice‐cold isolation buffer (225 mM mannitol, 75 mM sucrose, 1 mM EGTA, 5 mM HEPES‐KOH (pH 7.2) and 1 mg/mL of fatty‐acid‐free bovine serum albumin (BSA)). Homogenate was spun at 1100 *g* for 5 min at 4°C and supernatant was centrifuged (18 500 *g*) for 10 min at 4°C using a 15 vol% Percoll gradient. The pellet was resuspended in washing buffer (250 mM sucrose, 5 mM HEPES‐KOH (pH 7.2), 0.1 mM EGTA and 1 mg/mL of BSA) and centrifuged at 10000 *g* for 5 min at 4°C. The mitochondrial pellet was resuspended and protein concentrations determined using the Lowry method.

### 2.6 Mitochondrial Oxygen Consumption

Oxygen consumption and ΔΨm were simultaneously measured using a Clark‐type electrode (Oxytherm, Hansatech, UK) and a tetraphenylphosphonium (TPP^+^) ion selective electrode (World Precision Instruments, Sarasota, FL) during proton leak respiration as previously described [20]. Isolated cardiac ventricle mitochondria (0.1 mg) were added to 1‐mL of respiration buffer (200 mM sucrose, 25 mM KCl, 2 mM K_2_HPO_4_, 5 mM HEPES‐KOH (pH 7.2), 5 mM MgCl_2_, 0.2 mg/mL BSA) containing 80 ng/mL nigericin (to collapse ΔpH), 5 μM rotenone, 5 mM succinate, and oligomycin (2.5 μg/mL) at 37°C. ΔΨm was calculated using the Nernst equation [23]. In separate experiments, pyridoxal 5′‐phosphate (400 μM) was added to non‐specifically inhibit Aralar.

### 2.7 Steady‐State Electron Transport Chain Enzyme Complex Activities

Inhibitor sensitive ETC complex activities were measured in 1‐mL volume spectrophotometrically in isolated cardiac ventricle mitochondria [15, 24]. Rotenone‐sensitive Complex I specific activity was measured in cardiomyocyte mitochondria (40 μg) using 4.8 mM^−1^ cm^−1^ as the extinction coefficient of NADH at 340 nm with a reference wavelength of 380 nm. 2‐Thenoyltrifluoroacetone‐sensitive Complex II activity was measured in mitochondria (40 μg) using 19.1 mM^−1^ cm^−1^ as the extinction coefficient of 2,6‐dichlorophenolindophenol at 600 nm. For Complexes III and IV, inhibitor‐sensitive first‐order rate constants were calculated (4 μg and 2 μg mitochondria, respectively) using 18.5 mM^−1^ cm^−1^ as the extinction coefficient of cytochrome c at 550 nm. Oligomycin‐sensitive Complex V specific activity was measured in cardiomyocyte mitochondria (40 μg) with 6.2 mM^−1^ cm^−1^ as the extinction coefficient of NADH at 340 nm. Rotenone‐sensitive Complex I + III linked activity and antimycin A‐sensitive Complex II + III linked activity were measured separately in mitochondria (40 μg) with 18.5 mM^−1^ cm^−1^ as the extinction coefficient of cytochrome c at 550 nm.

## 3 Statistical Analysis

Statistical analysis was performed using GraphPad Prism 10 software (GraphPad Software, La Jolla, CA). Data was assessed for normality and are presented in the figures as means ± SD. The sample number of mice studied for each experiment is indicated for each figure. Sample size for each outcome measure was chosen to provide 80% power to detect an effect size of 15% between groups at a significance level of 0.05. Statistical tests utilized are delineated in each figure legend. Student's *t*‐test (two‐tailed) was used to assess for significance between two groups and one‐way analysis of variance (ANOVA) with Tukey's post hoc test was used to calculate significance between more than two groups. Significance was set at *p* < 0.05.

## 4 Results

### 4.1 Ubiquinone‐5 Induces Type 1 s‐Degree Atrioventricular Block

**FIGURE 1:**
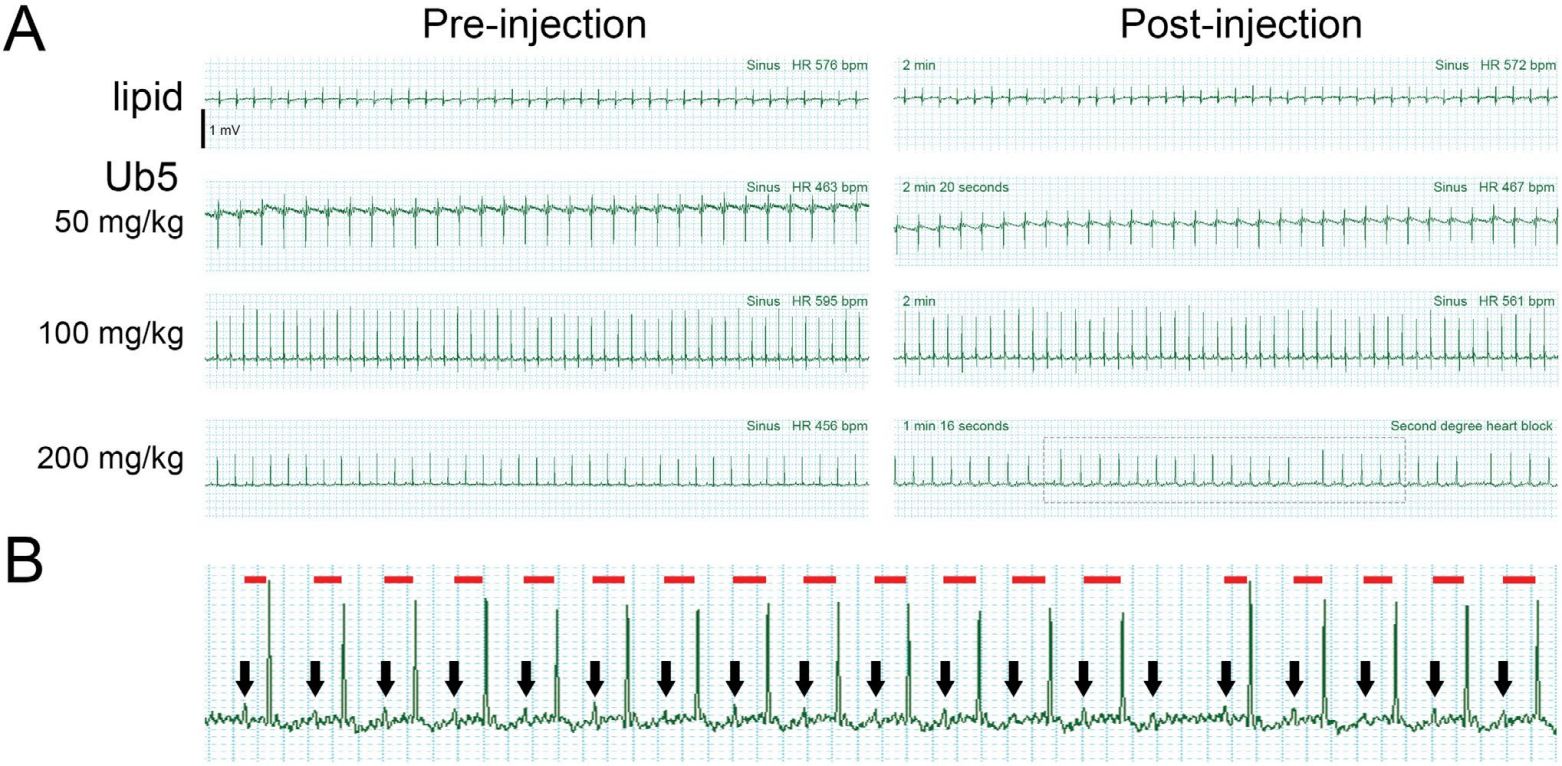
High dose Ubiquinone‐5 induces second‐degree AV block in vivo. Mice were injected separately with 3 different doses of Ub5 or intralipid vehicle control via tail vein. ECG was monitored continuously. (A) Representative ECG traces pre‐ and post‐injection from at least 3 animals per dosage. Time after injection is indicated. Boxed region highlights onset of second‐degree AV block following injection of 200 mg/kg Ub5. (B) Larger view of boxed region in (A). QRS complexes are clearly seen. Black arrows indicate P waves. Red lines indicate P‐R intervals. P‐R interval gradually prolongs prior to the AV block, indicating Wenckebach or Mobitz type 1 block.

**FIGURE 2:**
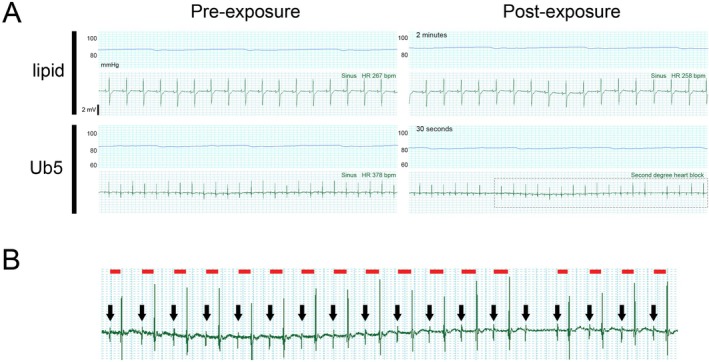
Ubiquinone‐5 causes second‐degree AV block ex vivo in the isolated‐perfused heart. Mouse hearts were exposed to Ub5 (200 mg per kg heart weight) or intralipid vehicle control. Surface ECG was monitored continuously. (A) Representative ECG traces pre‐ and post‐exposure from 3 animals per exposure. Time after injection is indicated. Boxed region highlights onset of second‐degree AV block in Ub5‐exposed heart. Aortic perfusion pressure (blue tracing) is depicted above each ECG trace. (B) Larger view of boxed region in (A). QRS complexes are clearly seen. Black arrows indicate P waves. Red lines indicate P‐R intervals. P‐R interval gradually prolongs prior to the AV block.

To determine how clinically relevant doses of Ub5 affect cardiac rhythm in vivo, we first assessed the electrocardiogram (ECG) in young adult mice following tail vein injection of Ub5. We evaluated 3 different doses of Ub5 in separate mice along with intralipid vehicle control. Ub5 doses were chosen to represent a subtherapeutic dose (50 mg/kg), a therapeutic dose (100 mg/kg, >ED_50_), and a relatively high dose, known to induce loss of the righting reflex in 100% of animals (200 mg/kg) [20]. There were no detectable effects of intralipid, 50 mg/kg Ub5, or 100 mg/kg Ub5 on cardiac rhythm in any injected mouse (Figure 1A). However, 200 mg/kg Ub5 induced second‐degree AV block shortly after injection (Figure 1A). Inspection of the P‐R intervals revealed a gradual prolongation prior to the AV conduction defect, indicating Wenckebach or Mobitz type 1 block (Figure 1B). The same rhythm disturbances were seen ex vivo in the isolated‐perfused young adult heart exposed to 200 mg Ub5 per kg heart weight, indicating a direct drug effect (Figure 2). Thus, a clinically relevant high dosage of Ub5 interfered with cardiac conduction. Of note, the perfusion pressure appeared to decrease slightly in the Ub5‐exposed heart, suggesting some degree of coronary vasodilation (Figure 2). However, we did not measure blood pressure in vivo in the mouse following injection; thus, it is unknown if hypoperfusion contributed to the onset of arrhythmia.

### 4.2 Ubiquinone‐5 Induces Excessive Proton Leak in Cardiomyocyte Mitochondria

**FIGURE 3:**
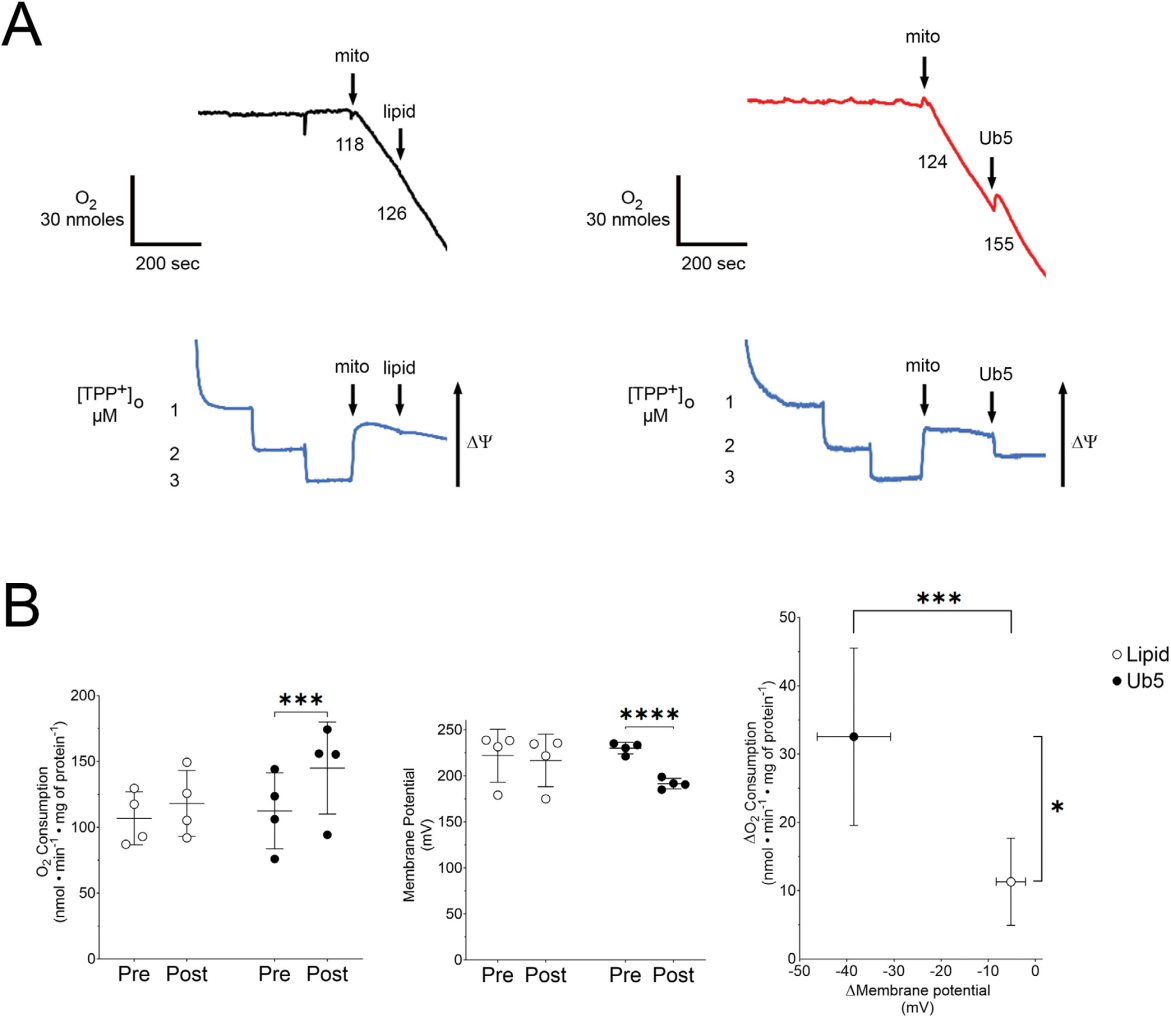
Ubiquinone‐5 induces excessive proton leak in cardiomyocyte mitochondria. Oxygen (O_2_) consumption and mitochondrial membrane potential (ΔΨm) were simultaneously measured during leak respiration in isolated mouse cardiomyocyte mitochondria (mito) exposed to lipid or Ub5 (200 μM). (A) Representative traces of O_2_ consumption (black or red) above with ΔΨm (blue) below. Numbers are O_2_ consumption rates (nmol•min^−1^•mg mitochondrial protein^−1^). ΔΨm was measured following tetraphenylphosphonium ion (TPP^+^) calibration. An increase in O_2_ consumption with a simultaneous fall in ΔΨm indicates an uncompensated increase in proton leak. (B) O_2_ consumption rates and membrane potential pre‐ and post‐exposure are depicted and ΔO_2_ consumption is graphed as a function of ΔMembrane potential. Data are means ± SD. *n =* 4 biological replicates from 4 different mice per group. *p* values were calculated using the Student's *t*‐test. ***p < 0.001, ****p < 0.0001.

Next, we exposed isolated cardiomyocyte mitochondria to Ub5 or equal volume of intralipid in vitro to determine mechanistic contributors of cardiotoxicity. We first assessed the effect on proton leak by quantifying and comparing changes in the rate of oxygen consumption and ΔΨm from baseline. Intralipid had little effect on the rate of respiration and ΔΨm (Figure 3). On the other hand, Ub5 caused a significant increase in leak respiration and a precipitous and significant decline in ΔΨm from baseline (Figure 3). Ub5‐induced changes in the rate of oxygen consumption and ΔΨm were significantly greater than intralipid‐induced changes (Figure 3B). Thus, Ub5 compromised ΔΨm due, in part, to an uncompensated increase in proton leak in isolated cardiomyocyte mitochondria.

### 4.3 Ubiquinone‐5 Inhibits Electron Transport at the Level of Coenzyme Q

**FIGURE 4:**
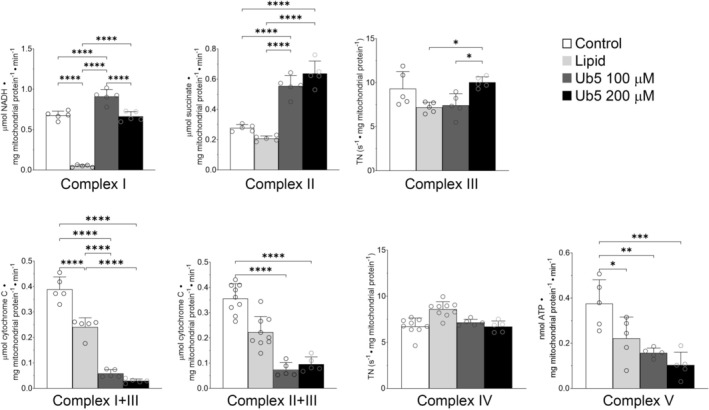
Ubiquinone‐5 inhibits the electron transport chain in cardiomyocyte mitochondria. Steady‐state kinetic activities were measured in non‐exposed controls and in isolated cardiomyocyte mitochondria exposed to lipid or Ub5. Specific activities for each enzyme complex are depicted. Coenzyme Q‐dependent electron transport was assessed by measuring linked Complex I + III and Complex II + III kinetic activities. First‐order Complexes III and IV rate constants were determined and expressed as turnover number (TN). Values are means ± SD. *n =* 5 mice per group. *p* values were calculated by one‐way ANOVA. *p < 0.05, ****p < 0.0001.

Next, we measured the kinetic activity of each of the electron transport chain (ETC) enzyme complexes during exposure to Ub5 or equal volume of intralipid in isolated mitochondria to determine where Ub5 interfered with the ΔΨm‐generating capacity. Intralipid significantly inhibited the steady‐state activities of Complex I, Complex I + III, and Complex V relative to unexposed controls (Figure 4). However, these effects were not functionally significant given the ability of lipid‐exposed mitochondria to generate an adequate ΔΨm (Figure 3). Ub5 stimulated the activities of Complexes I, II, and III relative to unexposed or lipid‐exposed controls depending on concentration, while inhibiting Complex V (Figure 4). The Ub5‐mediated effect on Complex V was not significantly different from the lipid effect (Figure 4). On the other hand, Ub5 significantly decreased the steady‐state linked activity of Complex I + III to a greater degree than lipid‐exposed mitochondria and significantly decreased Complex II + III activity relative to unexposed controls (Figure 4). Thus, the major perturbation induced by Ub5 was inhibition of the ETC at the level of coenzyme Q (given the effects on both Complex I + III and Complex II + III and the deviation from lipid‐mediated effects). Taken together, the data indicate that Ub5 induced uncompensated leak and inhibited the ETC at the level of coenzyme Q to interfere with the ability of cardiomyocyte mitochondria to generate and maintain an adequate ΔΨm.

### 4.4 Aralar Is the Source of Ub5‐Induced Leak and Is Targetable

**FIGURE 5:**
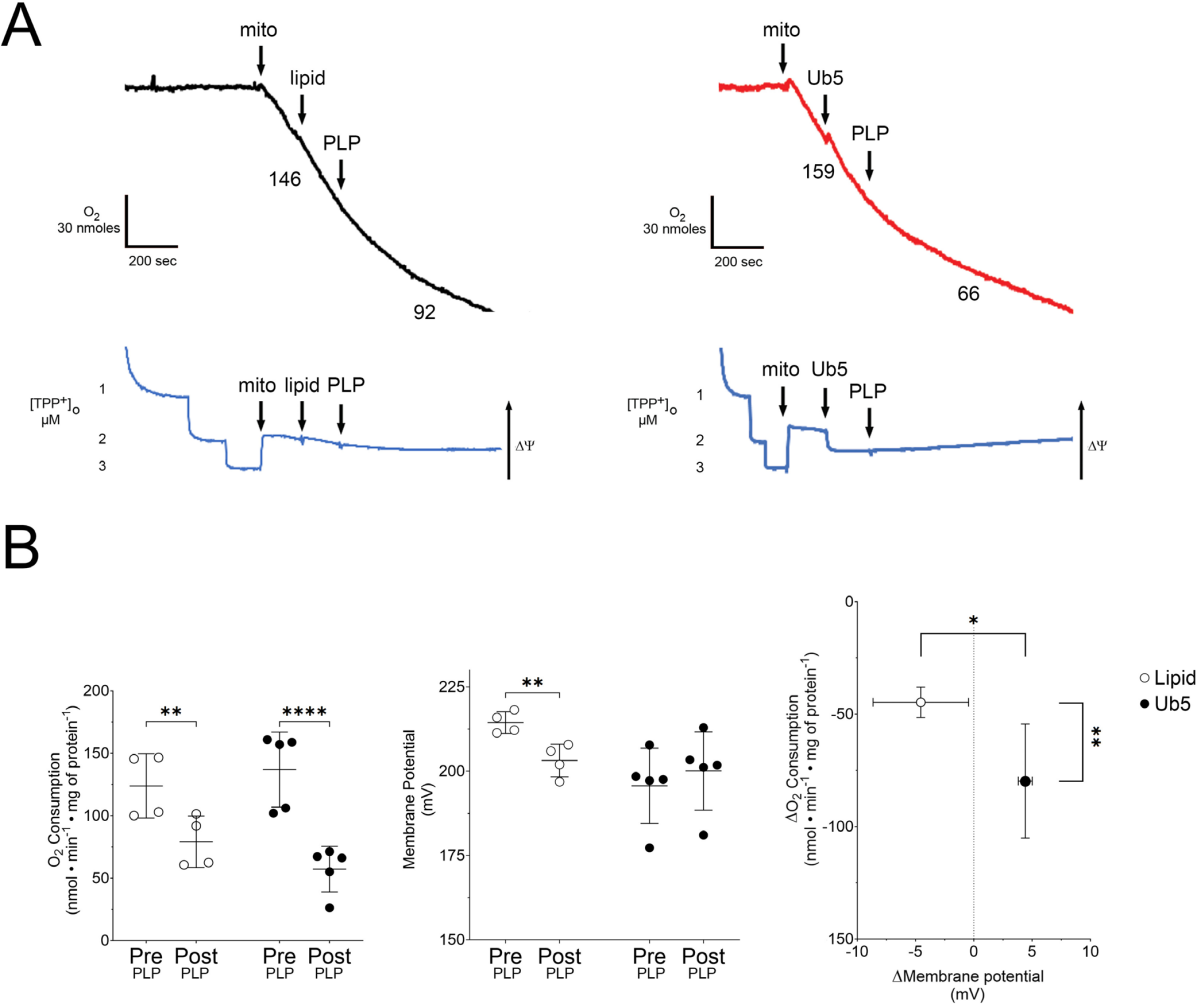
Pyridoxal 5′‐phosphate (PLP) blocks ubiquinone‐5‐induced proton leak. Oxygen (O_2_) consumption and mitochondrial membrane potential (ΔΨm) were simultaneously measured during leak respiration in isolated mouse cardiomyocyte mitochondria (mito) exposed to lipid or Ub5 (200 μM). (A) Representative traces of O_2_ consumption (black or red) above with ΔΨm (blue) below. Numbers are O_2_ consumption rates (nmol•min^−1^•mg mitochondrial protein^−1^). ΔΨm was measured following tetraphenylphosphonium ion (TPP^+^) calibration. A decline in O_2_ consumption with a rise in ΔΨm following addition of PLP indicates blockade of proton leak. (B) O_2_ consumption rates and membrane potential pre‐ and post‐PLP are depicted and ΔO_2_ consumption is graphed as a function of ΔMembrane potential. Data are means ± SD. *n =* 4–5 biological replicates from 4–5 different mice per group. *p* values were calculated using the Student's *t*‐test. *p < 0.05, **p < 0.01, ****p < 0.0001.

Next, we aimed to determine the role of the aspartate–glutamate carrier, Aralar, in Ub5‐induced leak in cardiomyocyte mitochondria given its identification as a functional target in forebrain mitochondria [20]. Using pharmacological blockade and genetic silencing we assessed for changes in the rate of oxygen consumption and ΔΨm during leak respiration in Ub5‐ and lipid‐exposed mitochondria. The non‐specific Aralar inhibitor, pyridoxal 5′‐phosphate (PLP), caused a significant decrease in leak respiration in Ub5‐exposed mitochondria along with a slow and steady rise in ΔΨm (Figure 5). In contrast, PLP caused significant declines in both the rate of leak respiration and ΔΨm in lipid‐exposed mitochondria, suggesting PLP‐mediated ETC inhibition (Figure 5). In separate experiments we confirmed that PLP does, indeed, inhibit the ETC to compromise ΔΨm in naïve mitochondria (Figure S1). The changes in oxygen consumption and ΔΨm post‐PLP differed significantly between lipid‐ and Ub5‐exposed mitochondria (Figure 5B). Importantly, the significant decline in leak respiration in combination with a relatively positive change in ΔΨm post‐PLP indicated inhibition of a major source of leak in Ub5‐exposed mitochondria (Figure 5).

**FIGURE 6:**
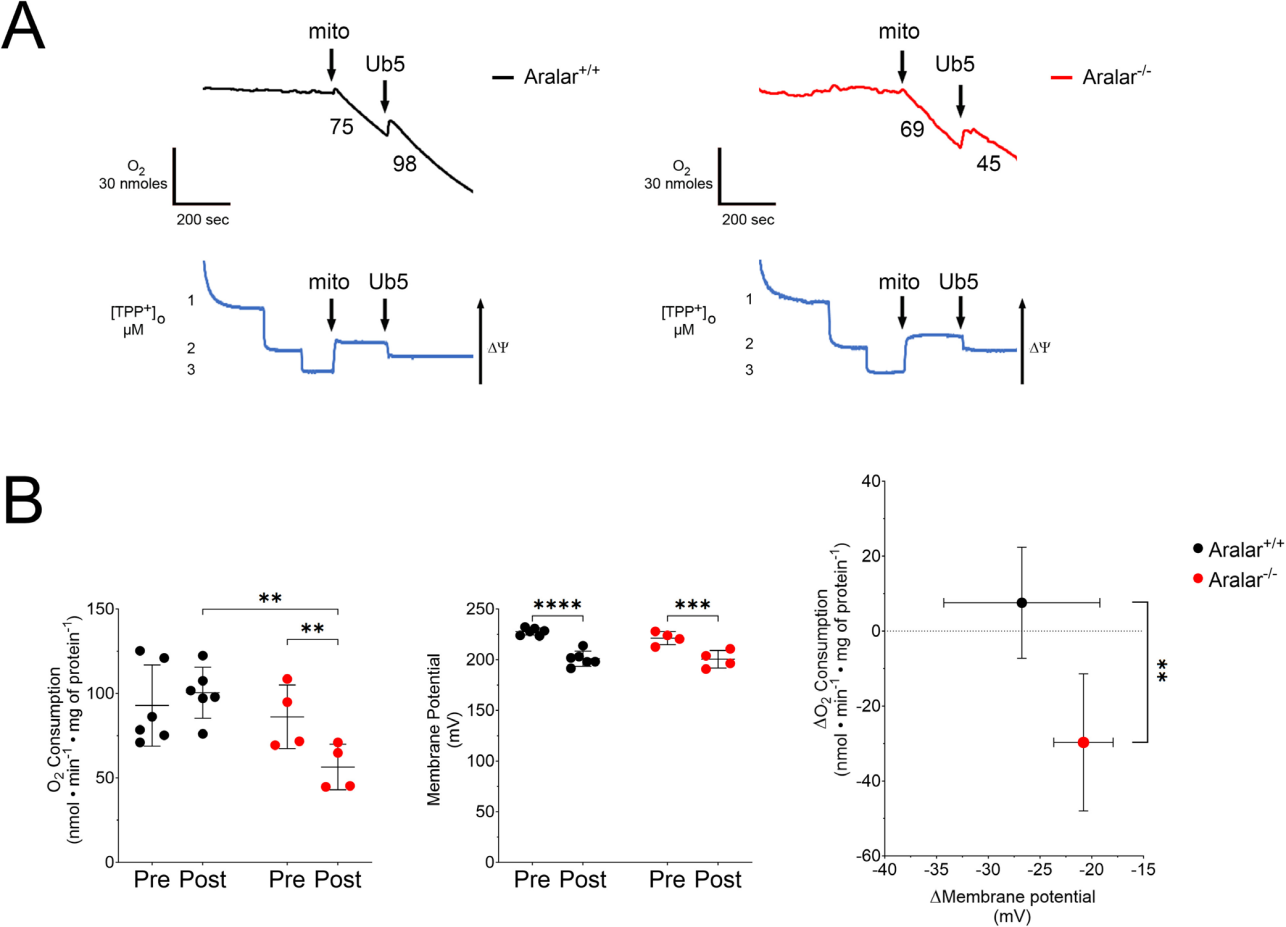
Ubiquinone‐5 fails to induce leak in Aralar‐deficient mitochondria. Oxygen (O_2_) consumption and mitochondrial membrane potential (ΔΨm) were simultaneously measured during leak respiration in cardiomyocyte mitochondria (mito) isolated from *Aralar* ^*−/−*^ mice and wild‐type littermate controls. (A) Representative traces of O_2_ consumption (black or red) above with ΔΨm (blue) below. Numbers are O_2_ consumption rates (nmol•min^−1^•mg mitochondrial protein^−1^). ΔΨm was measured following tetraphenylphosphonium ion (TPP^+^) calibration. An increase in O_2_ consumption with a fall in ΔΨm following addition of Ub5 (200 μM) indicates induction of excessive proton leak. (B) O_2_ consumption rates and membrane potential pre‐ and post‐Ub5 are depicted and ΔO_2_ consumption is graphed as a function of ΔMembrane potential. Data are means ± SD. *n =* 4–6 biological replicates from 4 *Aralar* ^*−/−*^ mice and 6 wild‐type littermate controls. *p* values were calculated using the Student's *t*‐test. **p < 0.01, ***p < 0.001, ****p < 0.0001.

Given that PLP is a non‐specific Aralar inhibitor, we next determined the effect of genetic silencing of Aralar on Ub5‐induced leak in isolated cardiomyocyte mitochondria. Ub5 caused a significant decline in ΔΨm in both *Aralar* ^*−/−*^ and wild‐type littermate control mitochondria without significant difference in the change in ΔΨm between groups (Figure 6). However, Ub5 failed to induce leak in *Aralar* ^*−/−*^ mitochondria, causing the rate of oxygen consumption to fall instead (Figure 6). Importantly, the change in the rate of respiration in mitochondria lacking Aralar differed significantly from wild‐type controls (Figure 6). The concomitant decline in the rate of oxygen consumption along with a fall in ΔΨm indicated Ub5‐mediated inhibition of the ETC in *Aralar* mutants; a process that was unmasked in the absence of excessive proton leak. These findings indicate that Aralar is the major source of Ub5‐induced leak.

**FIGURE 7:**
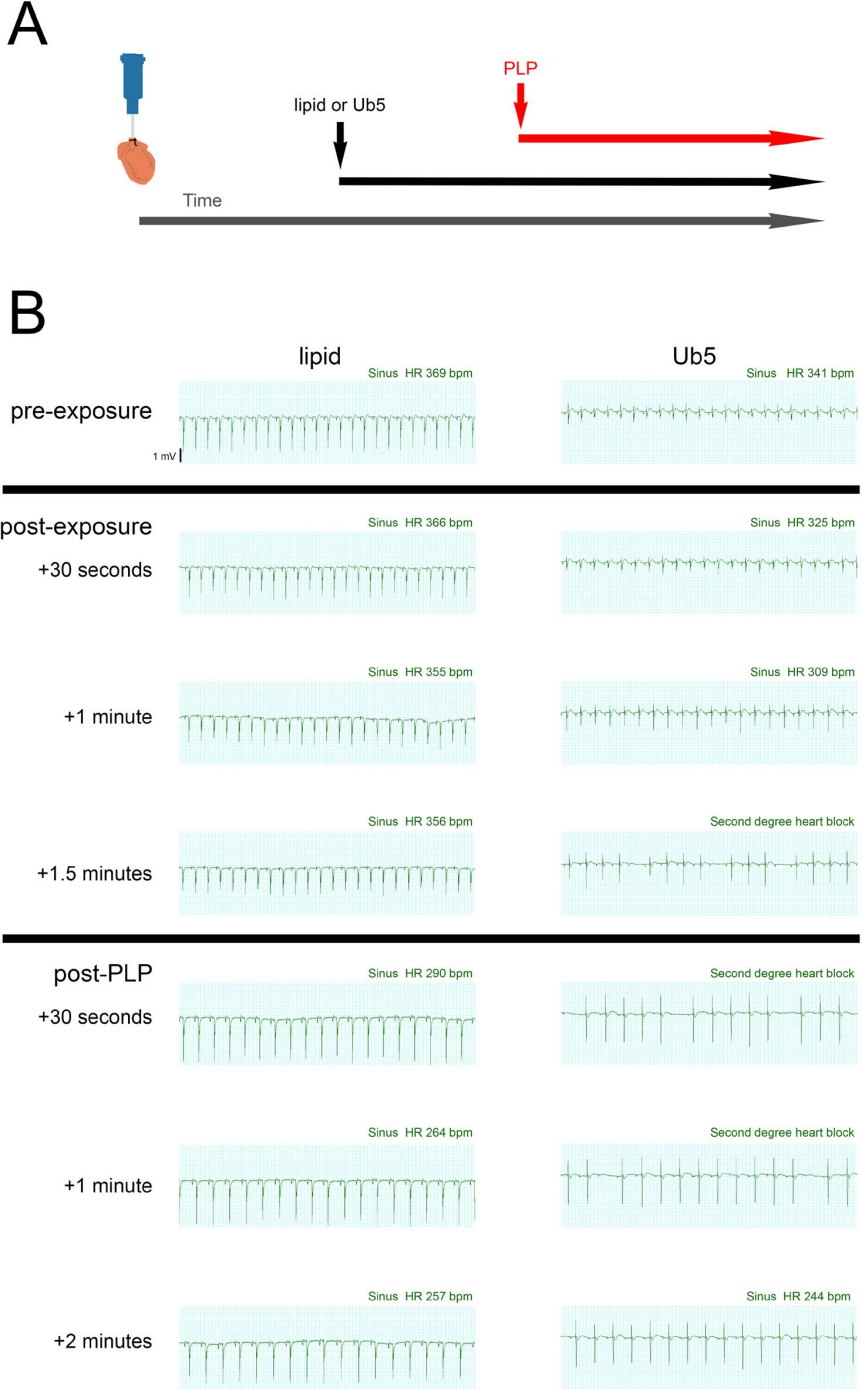
Pyridoxal 5′‐phosphate (PLP) rescues the isolated‐perfused heart from Ub5‐induced second degree AV block. Mouse hearts were exposed to Ub5 (200 mg per kg heart weight) or intralipid vehicle continuously. Surface ECG was monitored throughout. (A) Schematic of the experimental design. Ub5 or lipid exposure was initiated after stabilization. PLP was then added to the perfusate after onset of dysrhythmia in Ub5‐exposed hearts or at an equivalent timepoint in lipid‐exposed hearts. PLP was then continuously co‐administered with Ub5 or lipid. (B) Representative ECG traces pre‐ and post‐exposure to lipid, Ub5, and PLP from 3 animals per exposure.

Next, we aimed to determine the role of Aralar in Ub5‐induced second‐degree AV block. In prior work, we found that the *Aralar* ^*−/−*^ newborn heart was relatively resistant to the toxic effects of Ub5 on cardiac rhythm [20]. Unfortunately, *Aralar* ^*−/−*^ mice do not survive beyond a few weeks of life [25]. Therefore, we opted to target Aralar in the isolated‐perfused young adult wild‐type murine heart using pharmacological blockade with PLP. We assessed the effect of PLP on Ub5‐induced rhythm disturbances during continuous infusion using lipid as a vehicle control. Although there were no changes in cardiac rhythm in the isolated‐perfused intralipid‐exposed heart, we observed a decline in heart rate shortly after initiation of PLP (Figure 7). In separate experiments, we determined that PLP exposure slowed the spontaneous heart rate in naïve hearts (Figure S2). Such a chronotropic effect may relate to PLP‐mediated inhibition of the ETC. However, other mechanisms are possible. As with our initial experiments, we found that Ub5 rapidly induced second‐degree AV block in the isolated‐perfused heart (Figure 7). Exposing the heart to PLP after the onset of AV block restored the rhythm to sinus within minutes, albeit at a slower rate than baseline (Figure 7). The ability of PLP to rescue the isolated‐perfused heart from the Ub5‐mediated rhythm disturbance suggested that Aralar plays a functional role in Ub5‐induced cardiotoxicity.

## 5 Discussion

Anesthetic drugs are commonly used in clinical practice for their sedative‐hypnotic properties; however, many can adversely affect the heart in a dose‐dependent manner [3, 4, 5, 6, 7]. Here, we found that a clinically relevant high dosage of the anesthetic, Ub5, caused type 1 s‐degree heart block in the young adult mouse heart both in vivo and ex vivo. Several different medications are known to induce this type of arrhythmia, and Mobitz 1 s‐degree AV block has been described with use of the intravenous anesthetic, propofol [9, 11, 26]. Propofol depresses AV nodal conduction in a concentration‐dependent manner and prolongs the Wenckebach cycle length and effective refractory period [9]. These negative dromotropic effects are thought to be mediated by direct activation of the muscarinic M_2_ receptor [9]. However, propofol and Ub5 have different mechanisms of action within the central nervous system and, therefore, may not share molecular targets in the heart to elicit dysrhythmias [20].

We previously found the aspartate–glutamate carrier, Aralar, to be a pharmacological anesthetic target of Ub5 and source of uncompensated proton leak within forebrain mitochondria [20]. Activation of Aralar proved to be an important mechanistic contributor to the Ub5‐mediated anesthetic response [20]. However, this mechanism was specific to Ub5 given the lack of a functional effect of propofol on Aralar [20]. In the heart, genetic silencing of Aralar rendered the isolated‐perfused newborn mouse heart relatively resistant to toxic doses of Ub5 but not propofol [20]. Thus, Ub5 and propofol likely induce arrhythmias via different mechanisms. In the current work, we found that Ub5 compromised ΔΨm in isolated cardiomyocyte mitochondria by inhibiting electron transport at the level of coenzyme Q and inducing excessive proton leak via Aralar. Pharmacological Aralar inhibition rescued disturbances in cardiac rhythm in the isolated‐perfused young adult heart, indicating a functional role for Aralar‐mediated proton leak in Ub5‐induced arrhythmia. Thus, the adverse effects of Ub5 in the murine heart were on‐target given the known mechanism of Ub5‐induced unconsciousness [20].

ΔΨm is the major component of the proton motive force within mitochondria and is required for aerobic adenosine triphosphate (ATP) synthesis [27]. Defects in the ability of mitochondria to generate or sustain ΔΨm limit high energy phosphate production and jeopardize cellular homeostasis. In the heart, the majority of ATP is used to support contraction and relaxation and impaired bioenergetic capacity can result in heart failure [28]. However, mitochondrial defects resulting in impaired oxidative phosphorylation can also result in rhythm disturbances [29]. For example, patients with Kearns‐Sayre syndrome can develop complete heart block and those with Leigh syndrome can develop arrhythmias and conduction defects [30, 31].

Insight into the mechanistic connection between impaired mitochondrial function and dysrhythmias can be gleaned from *Ndufs4* ^−/−^ mice; a model for Leigh syndrome [32]. Mice harboring the global *Ndufs4* ^−/−^ knockout mutation have Complex I deficiency, compromised ΔΨm, and an increase in the NADH/NAD^+^ ratio [32, 33]. The shift in NADH/NAD^+^ leads to hyperacetylation of the cardiac sodium channel, Na_V_1.5, which reduces sodium current and results in bradyarrhythmias, second‐degree heart block, and rarely complete heart block [33]. Although we did not assess for Na_V_1.5 acetylation as part of this study, such a mechanism could be the link between a compromised ΔΨm and dysrhythmias and could explain how Ub5 induces second‐degree heart block.

We recognize that the current investigation has several limitations. For example, we did not determine the effect of Ub5 on other aspects of cardiac function, such as systolic contractile force or diastolic relaxation. Therefore, future work will need to assess such processes and will explore the cardiac effects of Ub5 in larger animal models. We also acknowledge that our assessment of dromotropy was crude, involving simple observation of changes in the P‐R interval during Ub5 exposure. Thus, we plan to quantify AV nodal conduction using a more in‐depth experimental approach in subsequent investigations. Furthermore, we appreciate the need to elucidate exact mechanisms of Ub5‐induced AV block (such as the role of the muscarinic M_2_ receptor or Na_V_1.5 acetylation), downstream from effects on ΔΨm. Finally, we did not formally assess for the effects of Ub5 on coronary perfusion. Since hypoperfusion can contribute to the onset of arrhythmias, evaluation of such will be necessary in future work.

Despite these limitations, our findings carry significance. First, the work demonstrates that, as with other anesthetics, Ub5 has cardiac side‐effects and can induce arrhythmias. Second, an upstream contributory mechanism of Ub5‐induced cardiotoxicity (i.e., Aralar activation) appears to be targetable. This latter property separates Ub5 from other sedative‐hypnotics and has implications for future drug development. As such, pharmacological inhibition of Aralar in the heart serves as a clinically relevant strategy to attempt to prevent Ub5‐mediated arrhythmias. Success and feasibility of this approach could render Ub5 safer as an anesthetic, with limited cardiac side‐effects. However, inability to block Aralar in the heart in vivo might prove to be an insurmountable roadblock, preventing further development of Ub5 as a viable sedative‐hypnotic. Regardless of the outcome of future work, the field should strive to develop new anesthetic agents with preventable cardiac side‐effects and limited cardiotoxicity. Discovery of such novel sedative‐hypnotics would be innovative and would advance the discipline of anesthesiology forward, enhancing safety and efficacy.

## Author Contributions

Conceptualization: H.L., R.J.L., methodology: H.L., R.L., C.S., R.J.L., investigation: H.L., R.L., C.S., R.J.L., data analysis: H.L., R.L., R.J.L., writing – original draft: R.J.L., writing – review and editing: H.L., R.L., C.S., R.J.L.

## Funding

This work was supported by HHS | NIH | National Institute of General Medical Sciences (NIGMS), R01GM148716.

## Conflicts of Interest

Patent pending WO/2022/192633, R.J.L. (inventor), Columbia University (institution). The authors declare no conflicts of interest.

## Supporting information


**Figure S1:** Pyridoxal 5′‐phosphate (PLP) inhibits the electron transport chain. Oxygen (O_2_) consumption and mitochondrial membrane potential (ΔΨm) were simultaneously measured during leak respiration in isolated mouse cardiomyocyte mitochondria (mito). Representative traces of O_2_ consumption (black) above with ΔΨm (blue) below. Numbers are O_2_ consumption rates (nmol•min^−1^•mg mitochondrial protein^−1^). ΔΨm was measured following tetraphenylphosphonium ion (TPP^+^) calibration. A decline in O_2_ consumption with a fall in ΔΨm following addition of PLP indicates inhibition of the electron transport chain.
**Figure S2:** Pyridoxal 5′‐phosphate (PLP) slows the spontaneous heart rate in isolated‐perfused naïve hearts. Mouse heart was exposed to PLP after stabilization. Surface ECG was monitored continuously. Representative ECG traces pre‐ and post‐exposure are depicted.

## Data Availability

All data are available in the manuscript or in the Supporting information.
