# Supplementary material for: Targetable Effects of the Anesthetic, Ubiquinone‐5, on Murine Cardiac Rhythm
Source: FASEB J. 2026 Feb 16;40(4):e71598. doi: 10.1096/fj.202504065RR (PMC12908110; doi:10.1096/fj.202504065RR)

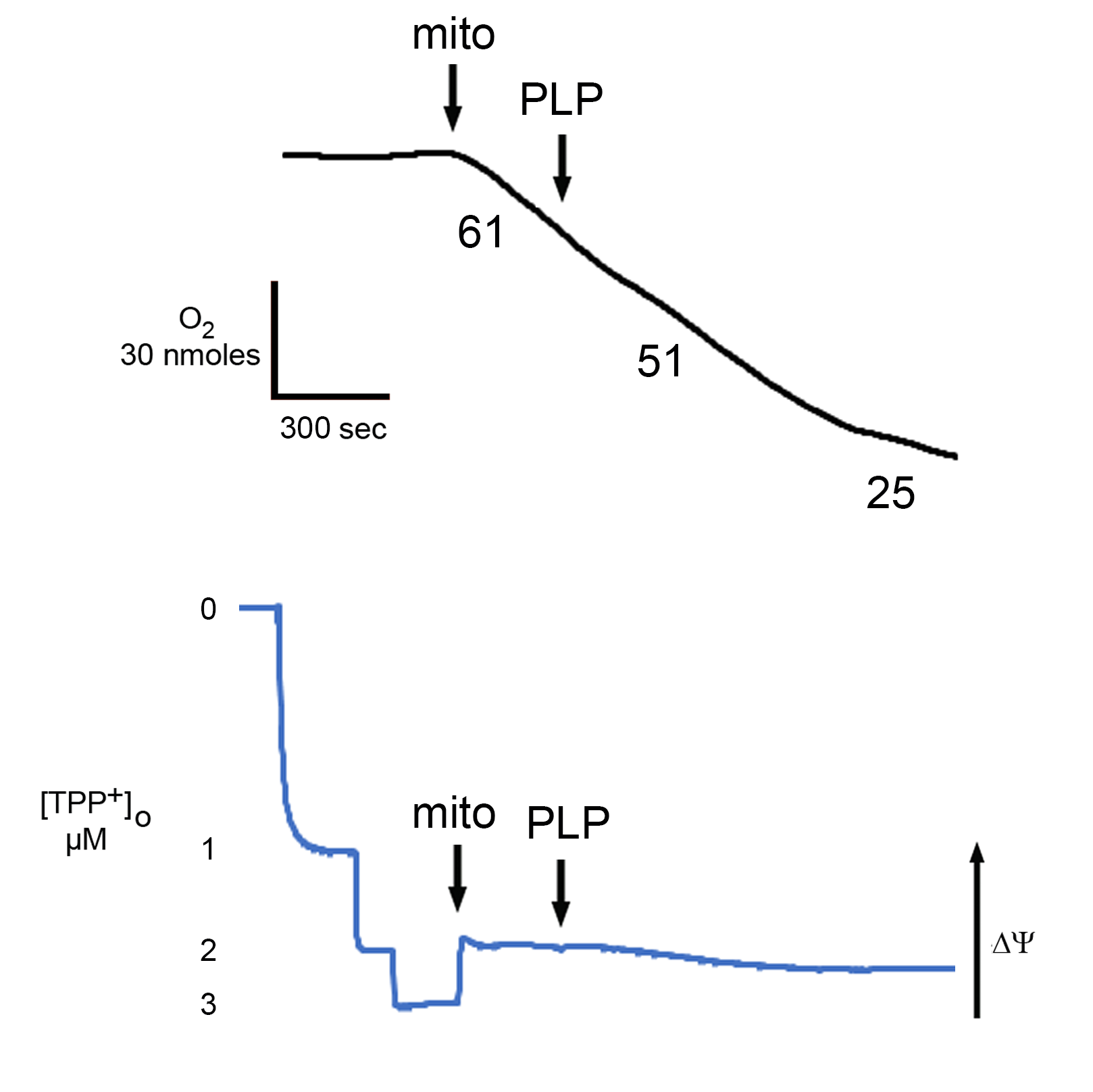


**Figure S1. Pyridoxal 5’-phosphate (PLP) inhibits the electron transport chain.** Oxygen (O_2_) consumption and mitochondrial membrane potential (ΔΨm) were simultaneously measured during leak respiration in isolated mouse cardiomyocyte mitochondria (mito). Representative traces of O_2_ consumption (black) above with ΔΨm (blue) below.  Numbers are O_2_ consumption rates (nmol•min^-1^•mg mitochondrial protein^-1^).  ΔΨm was measured following tetraphenylphosphonium ion (TPP^+^) calibration.  A decline in O_2_ consumption with a fall in ΔΨm following addition of PLP indicates inhibition of the electron transport chain.

**Figure S2. Pyridoxal 5’-phosphate (PLP) slows the spontaneous heart rate in isolated-perfused naïve hearts.** Mouse heart was exposed to PLP after stabilization. Surface ECG was monitored continuously. Representative ECG traces pre- and post-exposure are depicted.


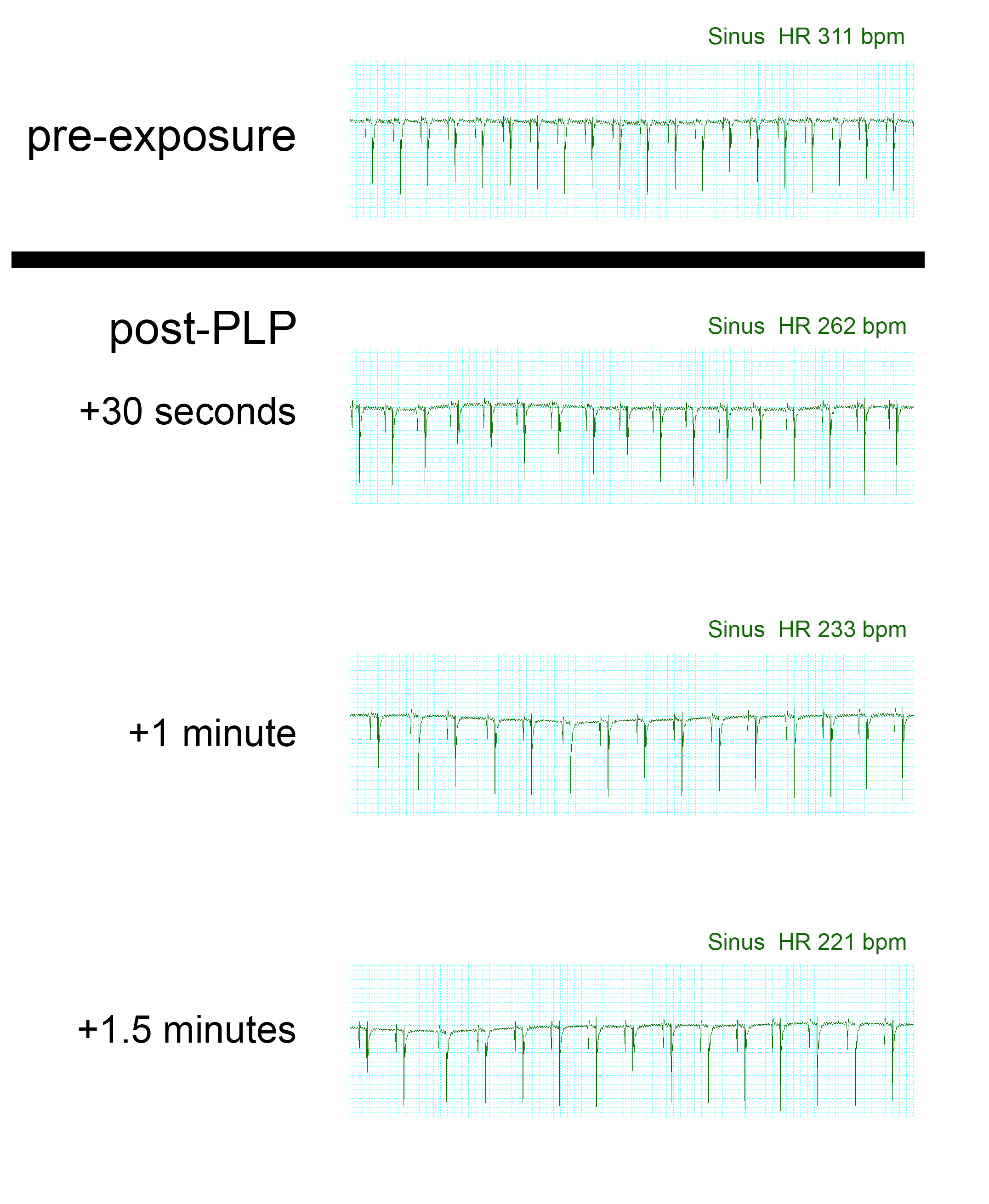

Supplement: Supplementary file 1 — Figure S1: Pyridoxal 5′‐phosphate (PLP) inhibits the electron transport chain. Oxygen (O2) consumption and mitochondrial membrane potential (ΔΨm) were simultaneously measured during leak respiration in isolated mouse cardiomyocyte mitochondria (mito). Representative traces of O2 consumption (black) above with ΔΨm (blue) below. Numbers are O2 consumption rates (nmol•min−1•mg mitochondrial protein−1). ΔΨm was measured following tetraphenylphosphonium ion (TPP+) calibration. A decline in O2 consumption with a fall in ΔΨm following addition of PLP indicates inhibition of the electron transport chain. Figure S2: Pyridoxal 5′‐phosphate (PLP) slows the spontaneous heart rate in isolated‐perfused naïve hearts. Mouse heart was exposed to PLP after stabilization. Surface ECG was monitored continuously. Representative ECG traces pre‐ and post‐exposure are depicted. [file FSB2-40-e71598-s001.docx]
